# Parents and friends both matter: simultaneous and interactive influences of parents and friends on European schoolchildren’s energy balance-related behaviours – the ENERGY cross-sectional study

**DOI:** 10.1186/1479-5868-11-82

**Published:** 2014-07-08

**Authors:** Saskia J te Velde, Mai JM ChinAPaw, Ilse De Bourdeaudhuij, Elling Bere, Lea Maes, Luis Moreno, Nataša Jan, Eva Kovacs, Yannis Manios, Johannes Brug

**Affiliations:** 1 Department of Epidemiology and Biostatistics and EMGO Institute for Health and Care Research, VU University Medical Center, P.O. Van der Boechorststraat 7, 1081 BT, Amsterdam, The Netherlands; 2 Department of Public and Occupational Health and EMGO Institute for Health and Care Research, VU University Medical Center, Amsterdam, The Netherlands; 3 Department of Movement and Sport Sciences, Ghent University, Ghent, Belgium; 4 Department of Public Health, Sport and Nutrition, University of Agder, Kristiansand, Norway; 5 Department of Public Health, Ghent University, Ghent, Belgium; 6 GENUD (Growth, Exercise, Nutrition and Development) Research Group. E.U. Ciencias de la Salud, Universidad de Zaragoza, 50009 Zaragoza, Spain; 7 Slovenian Heart Foundation, Ljubljana, Slovenia; 8 Department of Paediatrics, Pecs University, Pecs, Hungary; 9 Department of Nutrition and Dietetics, Harokopio University, Athens, Greece

**Keywords:** Parents, Friends, Social norm, Modelling, Rules, Soft drink, TV viewing, Physical activity

## Abstract

**Background:**

The family, and parents in particular, are considered the most important influencers regarding children’s energy-balance related behaviours (EBRBs). When children become older and gain more behavioural autonomy regarding different behaviours, the parental influences may become less important and peer influences may gain importance. Therefore the current study aims to investigate simultaneous and interactive associations of family rules, parent and friend norms and modelling with soft drink intake, TV viewing, daily breakfast consumption and sport participation among schoolchildren across Europe.

**Methods:**

A school-based cross-sectional survey in eight countries across Europe among 10–12 year old schoolchildren. Child questionnaires were used to assess EBRBs (soft drink intake, TV viewing, breakfast consumption, sport participation), and potential determinants of these behaviours as perceived by the child, including family rules, parental and friend norms and modelling. Linear and logistic regression analyses (n = 7811) were applied to study the association of parental (norms, modelling and rules) and friend influences (norm and modelling) with the EBRBs. In addition, potential moderating effects of parental influences on the associations of friend influences with the EBRBs were studied by including interaction terms.

**Results:**

Children reported more unfavourable friend norms and modelling regarding soft drink intake and TV viewing, while they reported more favourable friend and parental norms and modelling for breakfast consumption and physical activity. Perceived friend and parental norms and modelling were significantly positively associated with soft drink intake, breakfast consumption, physical activity (only modelling) and TV time. Across the different behaviours, ten significant interactions between parental and friend influencing variables were found and suggested a weaker association of friend norms and modelling when rules were in place.

**Conclusion:**

Parental and friends norm and modelling are associated with schoolchildren’s energy balance-related behaviours. Having family rules or showing favourable parental modelling and norms seems to reduce the potential unfavourable associations of friends’ norms and modelling with the EBRBs.

## Introduction

Overweight and obesity are important determinants of avoidable burden of disease [1,2] and overweight and obesity track from childhood into adulthood [3,4]. Preventing overweight and obesity and promoting healthy energy-balance related behaviours (EBRBs) in youth are therefore important health promotion priorities across Europe and beyond.

The family, and parents in particular, are considered the most important influencers regarding children’s EBRBs [5-9]. Parents can influence their children’s EBRBs through parental support and co-participation, demand or facilitation: parental EBRBs has been positively associated with children’s EBRBs, and parental rules, facilitation, co-participation have all been found to be associated with children’s EBRBs [10-14]. When children become older and gain more behavioural autonomy regarding different EBRBs, the parental influences may become less important and peer influences may gain importance [15,16]. During adolescence, children tend to spend more time with friends and children’s susceptibility to peer influences seems to peak around 12–13 years of age [17-19]. Therefore friends are likely to have increasingly more influence on the child’s behaviour during early adolescence through observation, modelling, imitation, companionship, social support, group norm setting, peer crowd affiliation and peer victimization [20-22]. A recent review [22] and several original studies indeed confirmed the influences of friends on physical activity behaviour [21,23-25] and eating behaviour [26,27]. The review by Salvy and colleagues showed that social facilitation or companionship and modelling are positively associated with both physical activity and eating; adolescents eat more and are more active in the presence of a peer or when peers eat more or are more active [22]. Contrary, Feunekes and colleagues found in one of the earlier studies on this topic that fat intake was not correlated between friends [28] and a review by Cunningham and colleagues concluded that there is no consistent evidence that friends influence body weight through their own weight-related behaviours [29].

**Figure 1:**
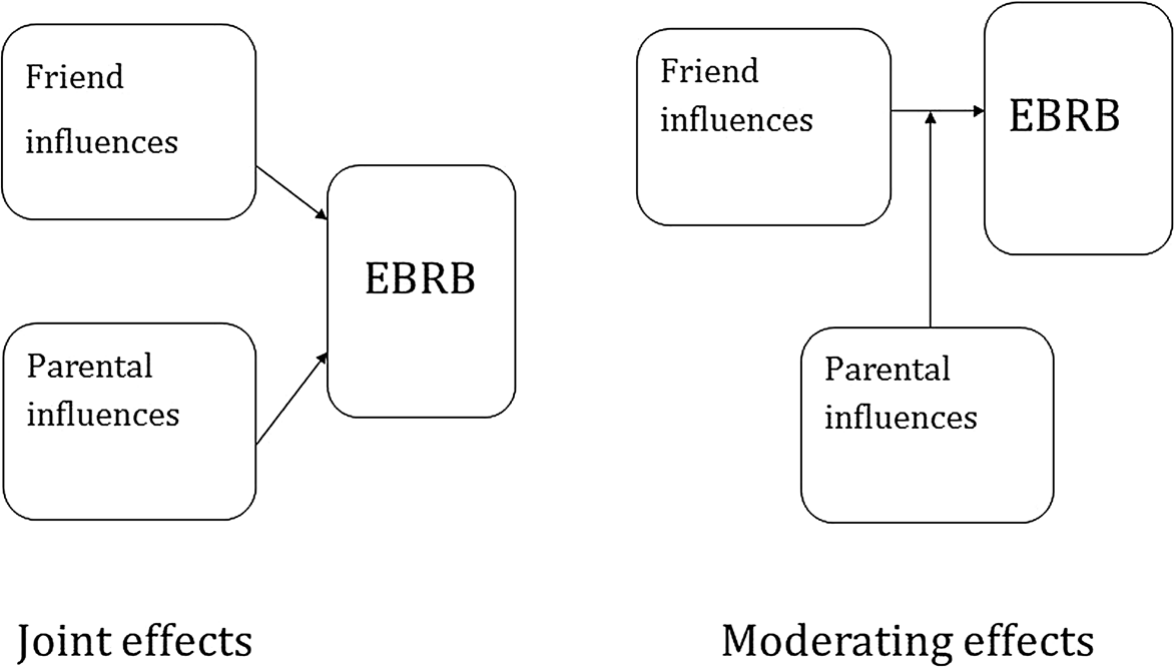
Conceptual model of the study, left panel represents a joint effect of parental and friend influences while the right panel represents an interaction or moderating effect of parental influences on the friend influences on energy balance-related behaviours (EBRBs).

Despite the likely increasing influence of peers on children’s behaviour when they become older, parental influences such as favourable parental norms, parental modelling and family rules remain important and may interact with the influence of peers, as depicted in Figure 1. Only a few studies investigated the parent and peer influences on EBRBs simultaneously [25,30-34]. In general, support from different sources is related to physical activity [30] and it may be that parents have a stronger influence on healthy food intake while friends have a stronger influence on unhealthy food intake [31,32]. However, to the best of our knowledge no studies investigated the potential interaction between parental and peer factors in their associations with children’s EBRBs.

Within the cross sectional study of the “EuropeaN Energy balance Research to prevent excessive weight Gain among Youth” (ENERGY)-project - a cross European study to explore overweight and its determinants in school children across Europe [35], schoolchildren reported the perceived peer norms and modelling with respect to four EBRBs (i.e. soft drink intake, TV viewing, breakfast consumption and physical activity), and also reported whether family rules were in place regarding these behaviours. Therefore, the present study assessed the associations of perceived peer influences (norms and modelling) and perceived parental influences (norms, modelling and rules) with schoolchildren’s EBRBs. Secondly, it assessed if parental influences regarding these behaviours have a moderating effect in the associations between peer influences and EBRBs. We are interested in favourable and unfavourable answers and associations, which we defined as having a presumed healthy or unhealthy influence (e.g. high modelling for TV viewing was seen as unfavourable, while high modelling for sport participation was seen as favourable).

We hypothesized that:

1. EBRB-specific family rules, parental and friend norms and modelling are all associated with the specific EBRBs (i.e. soft drink intake, TV viewing, daily breakfast consumption and sport participation), with more pronounced associations of parent influences on the healthy behaviours and more pronounced associations of friend influences on the unhealthy behaviours.

2. That favourable parental norms, modelling and rules regarding the specific EBRBs will reduce the potential unfavourable influence and/or strengthen the favourable influence of friend modelling and norms on the specific EBRBs.

## Methods

A description of the rationale and organization of the ENERGY-project [36] and a comprehensive description of the design, procedures, and methodology of the ENERGY school-based survey [35] are published elsewhere. The data collection manual and survey questionnaires for the Energy cross-sectional survey are available online at http://projectenergy.eu.

### Ethical approval

Ethical approval was obtained from the relevant Ethical review committees in all participating countries. Belgium the survey was approved by the Medical Ethics Committee of the University Hospital Ghent; in Greece the survey was approved by the Bioethics Committee of Harokopio University; in Hungary the survey was approved by the Scientific and Ethics Committee of Health Sciences Council; in the Netherlands the survey was approved by the Medical Ethics Committee of the VU University medical center; in Norway the survey was approved by the National Committees for Research Ethics in Norway; in Slovenia the survey was approved by the National Medical Ethics Committee of the Republic of Slovenia; and in Spain the survey was approved by Clinical Research Ethics Committee of the Government of Aragón. Furthermore, research permission was, if necessary, obtained from local school authorities (local school boards and/or headmasters).

### Sampling and respondents

The school-based survey was carried out between March and July 2010 in Belgium, Greece, Hungary, the Netherlands, Norway, Slovenia, and Spain, among pupils in the final years of primary education (aged 10–12 years). Based on previous cross-European studies on health behaviour and their determinants [37], a minimum sample of 1,000 schoolchildren per country and one parent/caretaker for each child was aimed for. Sampling was nationally representative in Greece, Hungary, the Netherlands, and Slovenia. In Spain, schools in the region of Aragón were selected; Belgium selected schools from Flanders and Norway selected schools from the southern regions of the country [35]. Within each country or region, three provinces were randomly selected from each of the lowest, mid and highest tertiles of degree of urbanization (i.e. the percentage of inhabitants living in towns of >20,000 persons). A municipality of >20,000 inhabitants from each selected province was randomly chosen, with schools randomly selected for inclusion in the study from all schools in that municipality. The clustering of the data was taken into account in sample size calculations. A school recruitment letter was sent to the headmaster or principal of the sampled schools, followed by a personal telephone call. Following the school’s agreement, parents received a letter explaining the study purpose and were asked for written consent for their child in countries where active informed consent (opt in) was required (Hungary, Norway, Spain) or were provided with a form to declare that their child was not to be included in the study in the other countries where ethical approval required passive informed consent (opt out).

Detailed information on response rates at the school, child and parent level have been reported elsewhere [38]. Between 15 (Slovenia) and 37 (Greece) schools participated, with a wide range in response rates at the school level. Response rates at the child level were in general high (>80%), but in Hungary (33%), Norway (45%) and Spain (43%) lower response rates were obtained, mainly because of parents not returning completed parental consent forms. For the current study we only used data from the child questionnaires. Only children who had valid data on at least one behaviour and the related friend and parental influence variables (norm, modelling, rules) were included in the analyses (n_total_ = 7811 (99%); n = 7698 (97%) for soft drink consumption; n = 7603 (96%) for TV viewing; n = 7674 (97%) for breakfast consumption; n = 7652 (97%) for sport/Physical Activity (PA) participation).

### Measures

Measurements were conducted according to standardized protocols. The children self-reported their engagement in the specific EBRBs and their perceptions of friend and parental norms and modelling and parental rules by means of questionnaires that were completed during school time. Detailed information regarding the procedures, training of research staff, the development of questionnaires [35], and test-retest reliability and construct validity of children and parent questionnaires [39,40] are published elsewhere. Test-retest reliability was tested by administering the questionnaire twice with a one week interval among 720 schoolchildren across the participating countries. The intraclass coefficients and percentage agreement was good to excellent for all items assessing the EBRBs. The intraclass coefficients and percentage agreement was good to excellent for 60% of items assessing parent and friend influences included in the current study (33;34). Construct validity of the questionnaire items was assessed by comparing the answers from the questionnaire to information retrieved through an interview [39]. This resulted in good to excellent construct validity (ICC > 0.60) for 63% of the items assessing the EBRBs. We also attempted to assess, construct validity for items assessing the perceived parent and friend influences regarding the EBRBs (33;34), with moderate to poor results, most likely due to lack of a clear gold standard.

#### Energy-balance related behaviours (EBRBs)

Sport-related physical activities, sedentary activities (TV-viewing and computer time), dietary behaviours (i.e. consumption of soft drinks, fruit juices, and breakfast) were assessed by self-report.

### Dietary behaviours

Intakes of soft drinks was assessed with food frequency questions (FFQ). Children were asked to indicate on how many days a week they normally drank soft drinks and on days that they drink soft drinks, how much by ticking the number of glasses (or small bottles, i.e. 250 ml), cans (i.e. 330 ml) and/or large bottles (i.e. 500 ml). Pictures of the serving sizes were shown in the questionnaire. Mean intake in ml of total soft drinks per day was calculated. However, intake of soft drinks was not normally distributed and was dichotomized for the analyses (based on a cut off value of 200 ml/day, which more or less equals one glass per day, and has been applied in this data set before).

Frequency of breakfast consumption was assessed by two questions asking the children on how many schooldays per week and on how many weekend days they normally had breakfast. Breakfast frequency per week was calculated by adding up the answers of the two questions. The frequency score was recoded into a daily breakfast score ([0] had breakfast 7 days/week; [1] had breakfast 0–6 times/week).

### Sport participation

questions assessed how many hours per week children participated in their two main sports. These questions have been used before and have shown good reliability and validity [41]. Children were first asked to mention their favourite sport with the instruction that we meant with sport ‘all sports activities that take place at a sports club and/or the supervision of a trainer/instructor/coach’, this could include both team and solitary sports activities. This question was followed by asking them about how often they did this sport in a normal week (with answer alternatives 30 minutes/week, 1 hour/week, 1.5 hours/week, 2 hours/week, 2.5 hours/week, 3 hours/week, 3.5 hours/week, 4 hours/week, 4.5 hours/week, and 5 hours/week). These questions were repeated for a second sport in which they participated. Children could also answer that they did not participate in any sport activity. Based on the answers, average time of sport participation per day was calculated for each child.

### TV viewing

Questionnaire items were included on how many hours the child normally watches TV (including video and DVD, but not including computer use and playing computer games) on weekdays and weekend days. Based on the answers, average time spent on TV viewing per day was calculated.

#### Friend norms, modelling and parental rules, norms and modelling

Friend norms were assessed by asking the children whether they thought that most of their friends would rate the specific EBRBs as good or bad (e.g. ‘If I drink soft drinks or fruit squash most of my friends think this is.... (very bad - very good)’).

Friend modelling was assessed by asking the child to rate the behaviour of their friends (e.g. ‘How often do most of your friends drink soft drinks or fruit squash? (Never - Always)’).

Parental rules as perceived by the child regarding the specific EBRBs were assessed by one question per EBRB (e.g. ‘Do your parents/caregivers have rules about how many soft drinks or fruit squash you are allowed to drink (No (0)- Yes (1))’).

Parental norms were assessed by questions asking how their parents would rate the specific EBRBSs (e.g. ‘If I drink soft drinks my parents think this is.....(very bad – very good)’).

Parental modelling was assessed by questions asking how often their parents engaged in the specific behaviour (e.g. how often do your parents drink soft drinks.....(never – always)).

All modelling and norm variables were recoded into categorical variables with three categories: 1. Never-not often; 2. Sometimes; 3. Often – always; or 1. Bad-very bad; 2. Neither good nor bad; 3. Good – very good.

### Statistical analyses

Means, standard deviations (SD) and proportions were calculated to describe the main variables. As descriptive results regarding the EBRBs [38] and perceptions of family and friend influences have been reported in previous publications [42,43], the current paper will only present these descriptive statistics to better understand the subsequent analyses.

Assumptions for linear regression analyses (e.g. normal distribution of the residuals) were checked. In case of skewed residuals, logistic regression was applied after dichotomizing the outcome variable (this was the case for soft drinks). Because of the nested design, i.e. children nested within schools, intraclass correlation coefficients (ICC) were calculated to estimate the strength of the clustering. ICCs were considered low (i.e. < 0.03), indicating that no adjustment for the nested design was needed. Therefore, (logistic) linear regression analyses were applied to estimate the associations of friend modelling and friend norms with the specific EBRBs. Regression coefficients or odds ratios (OR) and 95% Confidence Intervals (CI) were estimated to describe the strengths of the associations of parental and friend influences (i.e. norms and modelling) with the EBRBs. The norm and modelling variables were dummy coded before being entered into the regression models, with the neutral response (i.e. neither good nor bad; sometimes) as the reference category. For the analyses with TV viewing, the parental norm variable was dichotomized with the unfavourable category as the category of interest due to very few children giving ‘good – very good’ as the answer to this specific question. Also, for sport participation, perceived friend norm and modelling were dichotomized due to very few children giving ‘bad – very bad’ or ‘never – not often’ as an answer. However, here the unfavourable categories (‘bad – very bad’/‘never – not often’) could not be made the category of interest. In addition, associations of perceived parent norm with sport participation could not be estimated due to the fact that almost all children responded ‘good – very good’.

In order to study the potential moderating effect of parental influences (i.e. rules, norms, modelling), on the associations of friend norms and friend modelling with the EBRBs, interaction terms between parental influence variables and friend influence variables were calculated and entered into the regression model. If the interaction term approached statistical significance (p < 0.10), analyses were stratified. Predicted probabilities for high soft drink intake and daily breakfast consumption as well as predicted time spent watching TV or in sport were saved from the logistic and linear regression analyses and subsequently used for plotting the moderation effects.

All models were first run on the whole sample while adjusting for country, sex and age.

IBM SPSS statistics version 20 was used for the analyses.

## Results

The descriptive statistics in Table 1 show that the mean age in the total sample was between 11 and 12 years and that the distribution of boys and girls was almost equal. This table shows that the majority of the children reported to drink more than 200 ml/day of sugary drinks. Further, most children reported to eat breakfast daily. On average, children reported to spend 228 min/week on sports and 690 min/week on TV viewing (Table 1). More detailed country specific information can be found in a previously published article [38].

**Table 1:** Characteristics of the study population (N = 7811)

**Age (years)**	**Sex (boys)**	**Parental education (at least one parent > 14 years) (n = 5707)**	**Soft drink intake (ml/day)**	**Soft drink consumption (>200 ml/day)**	**TV viewing (min/week)**	**Breakfast consumption (daily)**	**Sport participation (min/week)**
**Mean (SD)**^**a**^	**N (%)**	**N (%)**	**Median**^**b**^**(IQR)**^**c**^	**N (%)**	**Mean (SD)**^**a**^	**N (%)**	**Mean (SD)**^**a**^
11.6 (0.7)	3747 (48)	3703 (64.9)	118.6 (35.7-462.9)	3329 (42.6)	767 (424)	5121 (65.6)	228.3 (166.3)

Country specific values are previously reported [38]; ^a^SD = standard deviation; ^b^Median is reported due to skewed distribution of the variable; ^c^IQR – Interquartile range (p25 – 75th percentile).

Table 2 shows the descriptive statistics regarding friend and parental influences. As reported before [Te Velde et al., Correlates paper, unpublished data], in general children reported unfavourable friend norm and modelling for the unhealthy behaviours (soft drinks and TV viewing) with 45% and 38% reporting that friends think that drinking soft drinks and TV viewing is ‘good – very good’ respectively. Less unfavourable parental norm and modelling for soft drink intake and TV viewing were reported by the children; with 13% and 8% reporting that parent would think that drinking soft drinks and TV viewing would be ‘good – very good’. About half of the children reported that rules were in place regarding soft drinks and TV viewing.

**Table 2:** **Descriptive statistics regarding friend norms and modelling, family rules, parent norms and modelling regarding soft drink intake, TV viewing, breakfast consumption and sport/physical activity participation for the whole sample**^**a**^

	**Behaviours**		**Soft drink intake**		**TV viewing**		**Breakfast consumption**		**Sport/physical activity participation**	
**Concept**	**Item**	**Response categories**	**n**	**%**	**n**	**%**	**n**	**%**	**n**	**%**
Friend norm	If I *< do specific behaviour >* my friends think this is.....	Bad - very bad	586	8%	644	8%	77	1%	32	0%
		Neither good nor bad	3665	47%	4133	54%	1270	16%	515	7%
		Good - very good	3514	45%	2885	38%	6409	83%	7235	93%
Friend modelling	how often do your friends *< specific behaviour >* ....	Never - not often	757	10%	226	3%	271	4%	182	2%
		Sometimes	3436	44%	1910	25%	1121	14%	1152	15%
		Often - always	3597	46%	5606	72%	6365	82%	6454	83%
Parent norm	If I *< do specific behaviour >* my parents think this is.....	Bad - very bad	2675	34%	2771	36%	40	1%	44	1%
		Neither good nor bad	4065	52%	4363	56%	288	4%	125	2%
		Good - very good	1050	13%	610	8%	7449	96%	7580	98%
Parent modelling	how often do your parents *< specific behaviour >* ....	Never - not often	3395	44%	1137	15%	533	7%	1693	22%
		Sometimes	3066	39%	3186	41%	833	11%	2475	32%
		Often - always	1328	17%	3447	44%	6413	82%	3593	46%
Family rules	Do your parents/caregivers have rules about….	Yes	4116	53%	4200	54%	3146	40%	2602	33%

^a^Country specific values are previously reported [38].

Regarding breakfast and sports/physical activity, children generally reported both favourable friend as well as parental norms and modelling. About 40% reported to have rules for breakfast consumption and about one third reported to have rules regarding sports/physical activity participation.

### Associations of friends norm and modelling with EBRBs

Perceived friend norms and modelling were significantly associated with the EBRBs (except favourable friend norm towards soft drink), so that unfavourable perceived friend norms and modelling were associated with more soft drink consumption, more TV viewing and lower odds for daily breakfast consumption. Similarly, favourable perceived friend norms and modelling were associated with lower soft drink intake and less TV time. After taking into account parental norms, modelling and family rules (Table 3) the associations weakened, but remained significant, except for the favourable friend norm category for TV time (Table 4) where the relationship lost statistical significance.

**Table 3:** Results of the (logistic) regression analyses for the associations of friend norm, friend modelling, parental norm, parental modelling and family rules with high soft drink intake, TV viewing, daily breakfast consumption and sport participation

		**Soft drink intake (> 200ml/day)**			**TV viewing (min/week)**			**Daily breakfast consumption**			**Sport participation (min/week)**		
		**OR**^**a**^	**95% CI**		**b**^**a**^	**95% CI**		**OR**^**a**^	**95% CI**		**b**^**a**^	**95% CI**	
Friend norm (If I *<do specific behaviour>* my friends think this is.....)	Bad- very bad	**0.66**	**.523**	**.833**	**-80.3**	**-116.0**	**-44.6**	**0.50**	**0.31**	**0.80**	**n.c.**^**b**^		
	Neither good nor bad	Reference			Reference			Reference			Reference		
	Good - very good	**1.67**	**1.50**	**1.86**	**97.0**	**75.7**	**118.4**				**44.0**	**29.7**	**58.4**
Friend modelling (How often do your friends *<specific behaviour>*....)	Never -not often	**0.54**	**0.44**	**0.67**	**-88.1**	**-146.2**	**-30.0**	**0.45**	**0.34**	**0.58**	n.c.^b^		
	Sometimes	Reference			Reference			Reference			Reference		
	Often - always	**2.04**	**1.83**	**2.27**	**129.0**	**106.5**	**151.4**				**36.1**	**26.0**	**46.1**
Parental norm (If I *<do specific behaviour>* my parents think this is.....)	Bad- very bad	**0.51**	**0.46**	**0.58**	Reference			**0.32**	**0.16**	**0.65**	n.c.^b^		
	Neither good nor bad	Reference						Reference			n.c.^b^		
	Good - very good	**2.00**	**1.71**	**2.34**	**148.4**	**112.5**	**184.3**				n.c.^b^		
Parental modelling (how often do your parents *<specific behaviour>*....)	Never -not often	**0.39**	**0.35**	**0.44**	Reference			**0.38**	**0.34**	**0.44**	**-25.1**	**-35.2**	**-15.0**
	Sometimes	Reference						Reference			Reference		
	Often - always	**2.29**	**1.98**	**2.65**	**180.2**	**161.2**	**199.2**				**36.4**	**28.2**	**44.5**
family rules (Do your parents have rules about….)	No	Reference			Reference			Reference			Reference		
	Yes	**0.60**	**0.54**	**0.66**	**-166.5**	**-185.5**	**-147.5**	**1.71**	**1.54**	**1.90**	**24.2**	**16.5**	**31.8**

^a^all models are adjusted for sex, age and country; **Bold** – significant at p < 0.05; ^b^ n.c – not calculated (due to very small group(s)).

**Table 4:** Results of the multiple (logistic) regression analyses for the associations of friend norm, friend modelling, parental norm, parental modelling and family rules, adjusted for each other, with high soft drink intake, TV viewing, daily breakfast consumption and sport participation

		**Soft drink intake(>200 ml/day)**			**TV viewing (min/week)**			**Daily breakfast consumption**			**Sport participation (min/week)**		
		**OR**^**a**^	**95% CI**		**b**^**a**^	**95% CI**		**OR**^**a**^	**95%CI**		**b**^**a**^	**95% CI**	
Friend norm (If I *< do specific behaviour >* my friends think this is.....)	Bad- very bad	0.89	0.69	1.15	**−42.3**	**−77.0**	**−7.60**	0.74	0.44	1.25	an.c.^b^		
	Neither good nor bad	Ref ^c^			Ref			Ref			Ref		
	Good - very good	**1.22**	**1.08**	**1.37**	**50.9**	**29.0**	**72.8**				**30.6**	**15.7**	**45.5**
Friend modelling (How often do your friends *< specific behaviour >* ....)	Never -not often	**0.66**	**0.53**	**0.82**	**−71.4**	**−128.8**	**−14.1**	**0.50**	**0.38**	**0.66**	n.c.		
	Sometimes	Ref			Ref			Ref			Ref		
	Often - always	**1.70**	**1.51**	**1.91**	**74.3**	**51.3**	**97.4**				**27.8**	**17.6**	**38.0**
Parental norm (If I *< do specific behaviour >* my parents think this is.....)	Bad- very bad	**0.67**	**0.59**	**0.76**	**Ref**			**0.39**	**0.18**	**0.85**	n.c.		
	Neither good nor bad	Ref						**Ref**			n.c.		
	Good - very good	**1.42**	**1.19**	**1.68**	**59.5**	**23.1**	**95.9**				n.c.		
Parental modelling (how often do your parents *< specific behaviour >* ....)	Never -not often	**0.46**	**0.41**	**0.52**	Ref			**0.41**	**0.36**	**0.47**	**−21.0**	**−31.3**	**−10.8**
	Sometimes	Ref						Ref			Ref		
	Often - always	**1.93**	**1.65**	**2.25**	**135.2**	**115.6**	**154.7**				**33.6**	**25.4**	**41.8**
family rules (Do your parents/caregivers have rules about….)	No	Ref			Ref			Ref			Ref		
	Yes	**0.74**	**0.67**	**0.83**	**−146.2**	**−165.1**	**−127.4**	**1.64**	**1.47**	**1.82**	**20.5**	**12.8**	**28.2**

^a^all models are additionally adjusted for sex, age and country; **Bold** – significant at p < 0.05; ^b^n.c – not calculated (due to very small group(s)); ^c^ref – reference category.

#### Associations of parental norms, modelling and rules with EBRBs

Parental norms, modelling and family rules were all significantly associated with all four EBRBs in a way that favourable norms and modelling were associated with lower soft drink consumption and more time spent in sports. Similarly, unfavourable parental norms and modelling were associated with more soft drink intake, more TV time, lower odds for daily breakfast and less time spent on sports. Having rules in place was associated with lower soft drink consumption and lower TV time and with higher odds for daily breakfast and more sport participation (Table 3). All aforementioned associations weakened but remained significant after adjusting for friend norms and friend modelling (Table 4).

#### Moderation by family rules with friend norms and friend modelling

**Figure 2:**
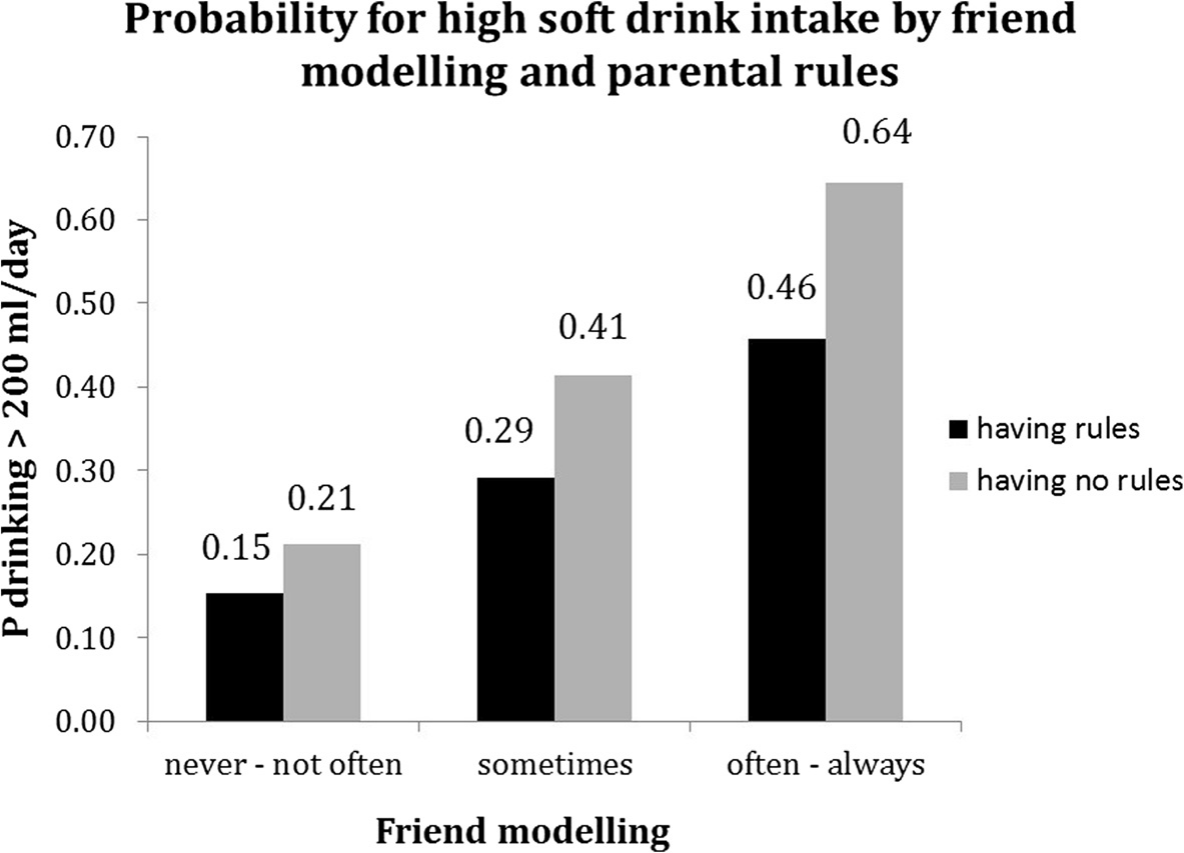
Moderation by parental rules in associations between friend modelling and soft drink intake.

**Figure 3:**
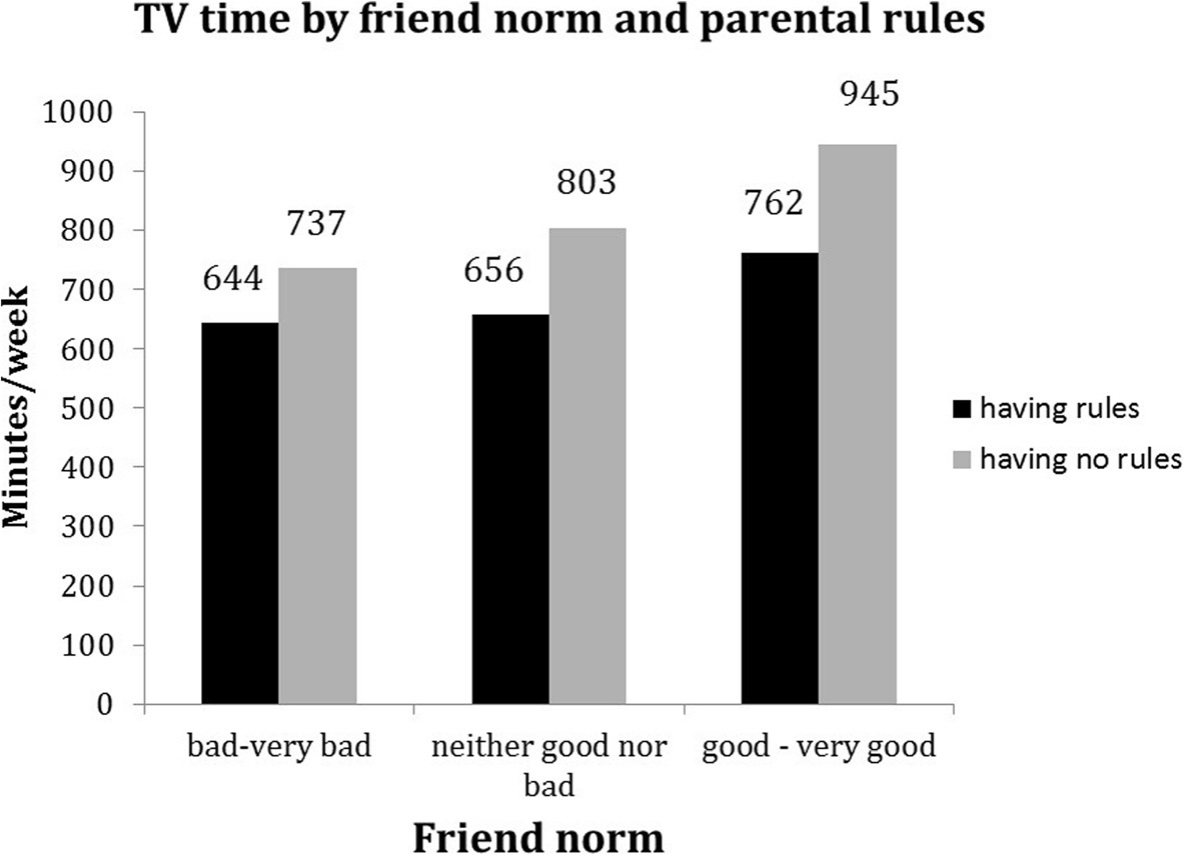
Moderation by parental rules in associations between friend norm and TV time.

**Figure 4:**
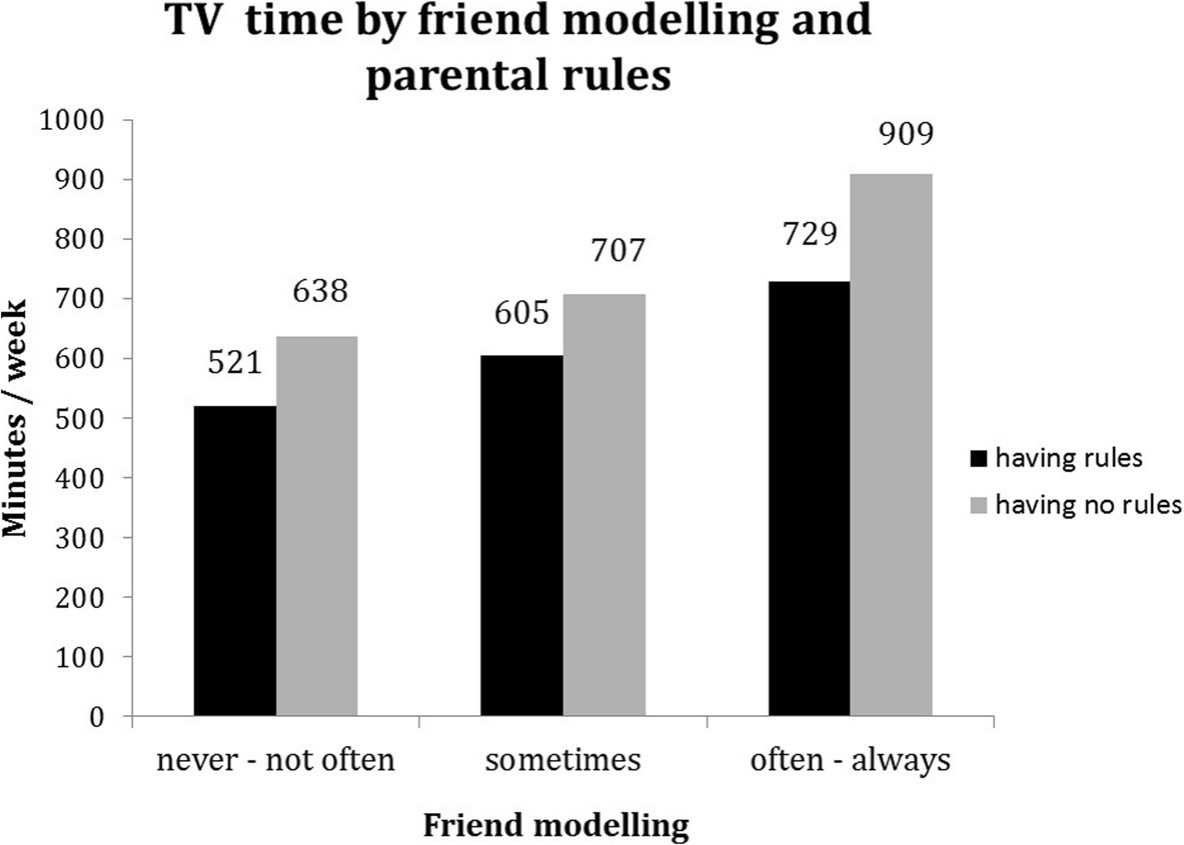
Moderation by parental rules in associations between friend modelling and TV time.

**Figure 5:**
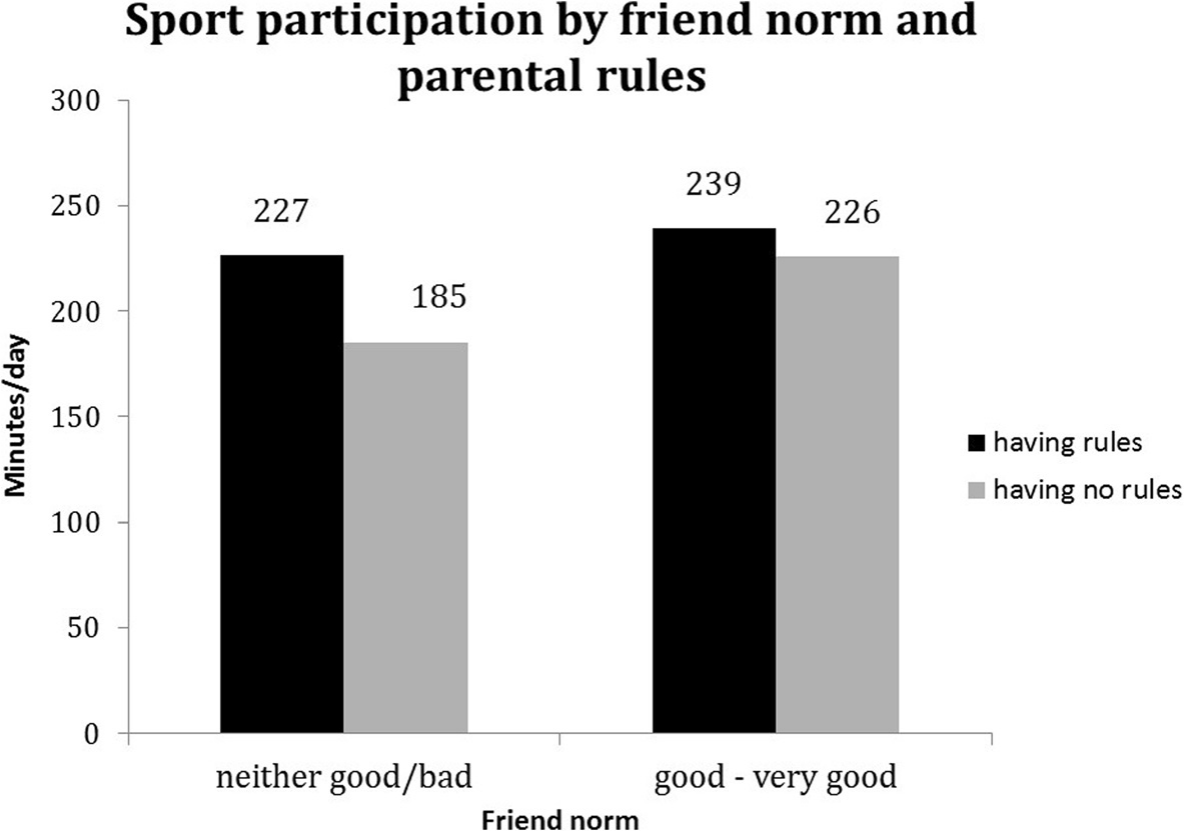
Moderation by parental rules in associations between friend norm and sport participation.

**Figure 6:**
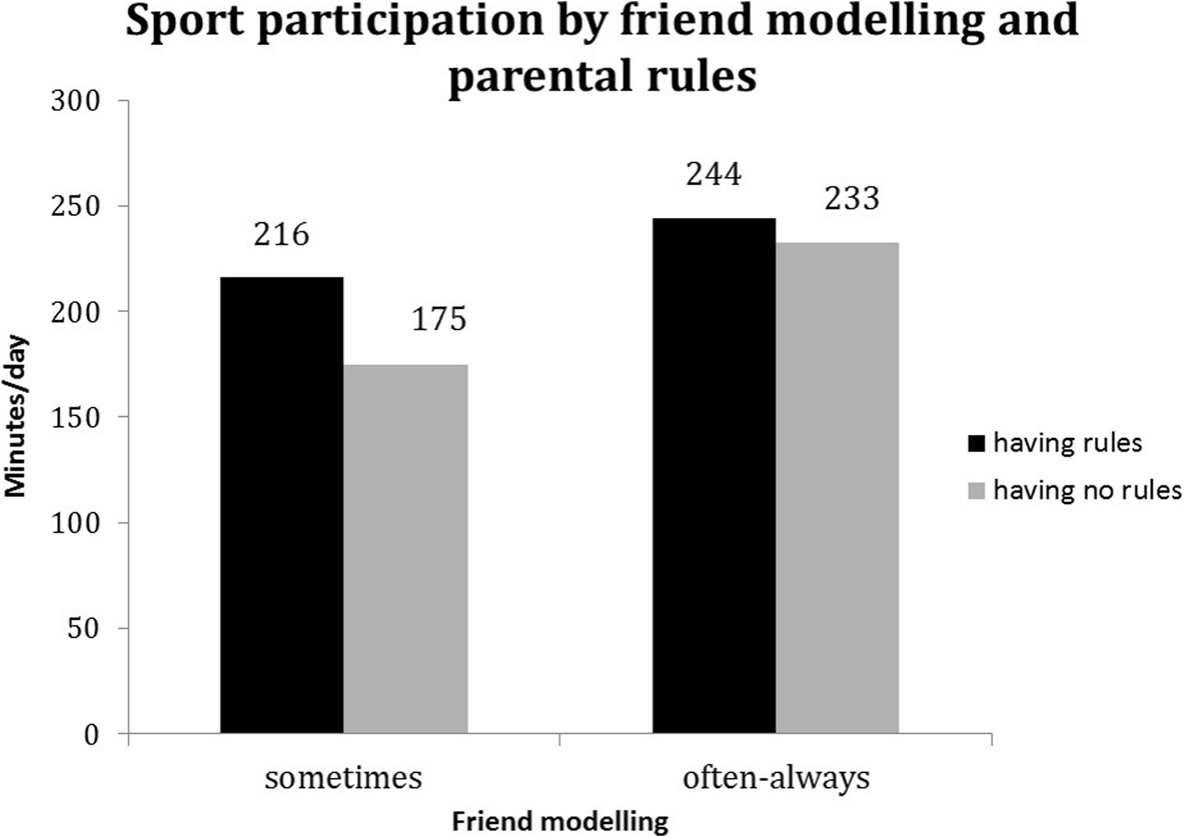
Moderation by parental rules in associations between friend modelling and sport participation.

For all behaviours except breakfast consumption, significant (p < 0.1) interaction terms with family rules were found (see Table 5). Although associations of friend norm and friend modelling with soft drink intake, TV viewing and sport participation were (borderline) significant in both strata, the difference between favourable and unfavourable friend norm and friend modelling appeared to be stronger in the absence of family rules (Table 5). In addition, Figures 2, 3 and 4 show that the highest probability for high soft drink intake and highest TV time was found in the absence of family rules (grey bars) combined with unfavourable friend norms and modelling. Whereas sport participation was highest when family rules were in place (black bars) combined with favourable friend norms and friend modelling (Figure 5 and 6).

**Table 5:** **Associations of friend norm and friend modelling with soft drink intake, TV viewing, daily breakfast and sport participation stratified**^**a **^**by family rules, parental norm and parental modelling**

		**Soft drink intake (>200ml/day)**				**TV viewing (min/week)**				**Sport participation (min/week)**			
**Parental rules in place**	**Friend norm**	**P-value interaction term**	**OR**^**b**^	**95% CI**		**P-value interaction term**	**b**^**b**^	**95% CI**		**P-value interaction term**	**b**^**b**^	**95% CI**	
**No**	Bad- very bad		-			**0.079**	**−117.2**	**−181.8**	**−52.7**		n.c.^c^		
	Neither good nor bad		-				Ref^d^				Ref		
	Good - very good		-			**0.059**	**107.2**	**74.1**	**140.3**	**0.070**	**53.1**	**36.2**	**70.0**
**Yes**	Bad- very bad		-				**−42.1**	**−82.6**	**−1.6**		n.c.		
	Neither good nor bad		-				Ref				Ref		
	Good - very good		-				**81.5**	**54.7**	**108.3**		20.3	−4.57	45.2
	**Friend modelling**												
**No**	Never -not often	0.188	**.48**	**.34**	**.66**	0.878	−91.1	−187.2	5.1		n.c.		
	Sometimes	**0.077**	Ref				Ref				Ref		
	often - always	0.149	**2.16**	**1.85**	**2.52**	**0.001**	**163.3**	**128.3**	**198.4**	**0.003**	**48.1**	**36.3**	**59.8**
**Yes**	Never -not often		**.60**	**.46**	**.80**		**−78.9**	**−148.0**	**−9.7**		n.c		
	Sometimes		Ref				Ref				Ref		
	Often - always		**1.87**	**1.60**	**2.17**		**95.8**	**67.9**	**123.7**		**20.1**	**4.11**	**36.1**
**Parental norm**	**Friend modelling**												
**‘Very bad -neither good nor bad’**	Never -not often					**0.009**	.96	−81.8	83.7				
	Sometimes						Ref						
	Ooften - always					0.281	**136.1**	**107.4**	**164.8**				
**‘Good - very good’**	Never -not often						**−145.2**	**−225.5**	**−65.0**				
	Sometimes						Ref						
	Often - always						**110.3**	**75.0**	**145.6**				
**Parental modelling**	**Friend modelling**	p-value for interaction term	b^b^	95% CI		**friend modelling**	p-value for interaction term						
**‘Never - sometimes’**	Never -not often	**<0.001**	**−147.6**^**e**^	**−210.1**	**−85.2**	**‘never - not often’**	never -not often	**0.035**	.68	.44	1.06		
	Sometimes		Ref^e^				sometimes - always		Ref				
	Often - always	**<0.001**	**62.7**^**e**^	**37.1**	**88.4**								
**‘Often - always’**	never -not often		119.9	−3.7	243.5	**‘Sometimes – always’**	never -not often		**.42**^**f**^	**.30**	**.58**		
	sometimes		Ref				sometimes - always		Ref^f^				
	often - always		**152.7**	**108.4**	**197.0**								

^a^stratified analyses were only conducted in case of significant interaction terms between parent and peer influences; ^b^ all models are adjusted for sex, age and country; **Bold** – significant at p < 0.05 for associations, p < 0.1 for interaction terms; ^c^n.c – not calculated (due to very small group(s)); ^d^ref – reference category; ^e^the parental modelling categories ‘never – not often’ and ‘sometimes’ were merged; ^f^the parental modelling categories ‘sometimes’ and ‘often-always’ were merged.

#### Moderation by parental norms with friend norms and modelling

**Figure 7:**
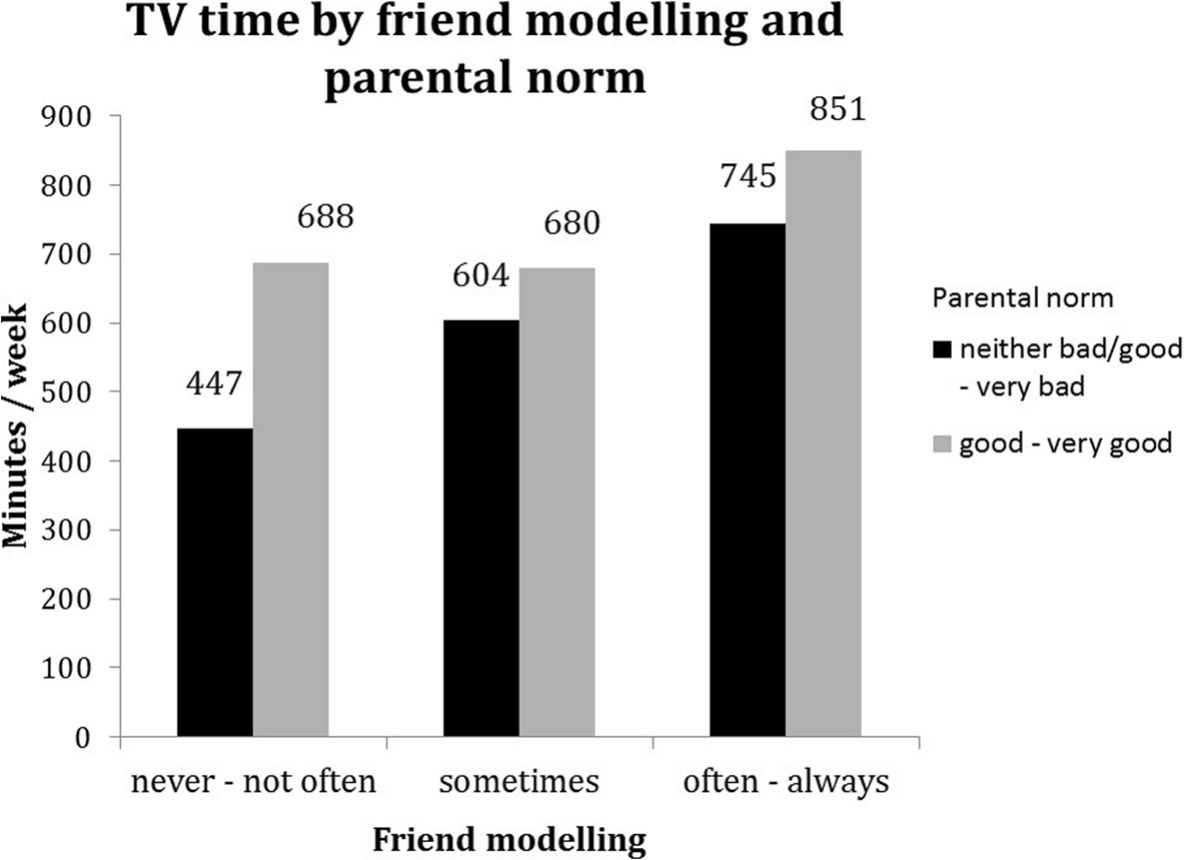
Moderation by parental norms in the associations between friend modelling and TV time.

One significant interaction term indicated that the strength of the association of friend modelling with TV time was dependent on parental norm (see Table 5). Results suggest that high, thus unfavourable, friend modelling had the strongest association with TV time in the favourable parental norm stratum (‘very bad – neither good nor bad’) (Table 5), but highest TV time was reported in the combination of unfavourable parental norm (grey bars) and high friend modelling (Figure 7).

#### Moderation by parental modelling with friend norms and modelling

Significant interaction terms were found for parental modelling with friend modelling, and stratified analyses were conducted as shown in Table 5.

**Figure 8:**
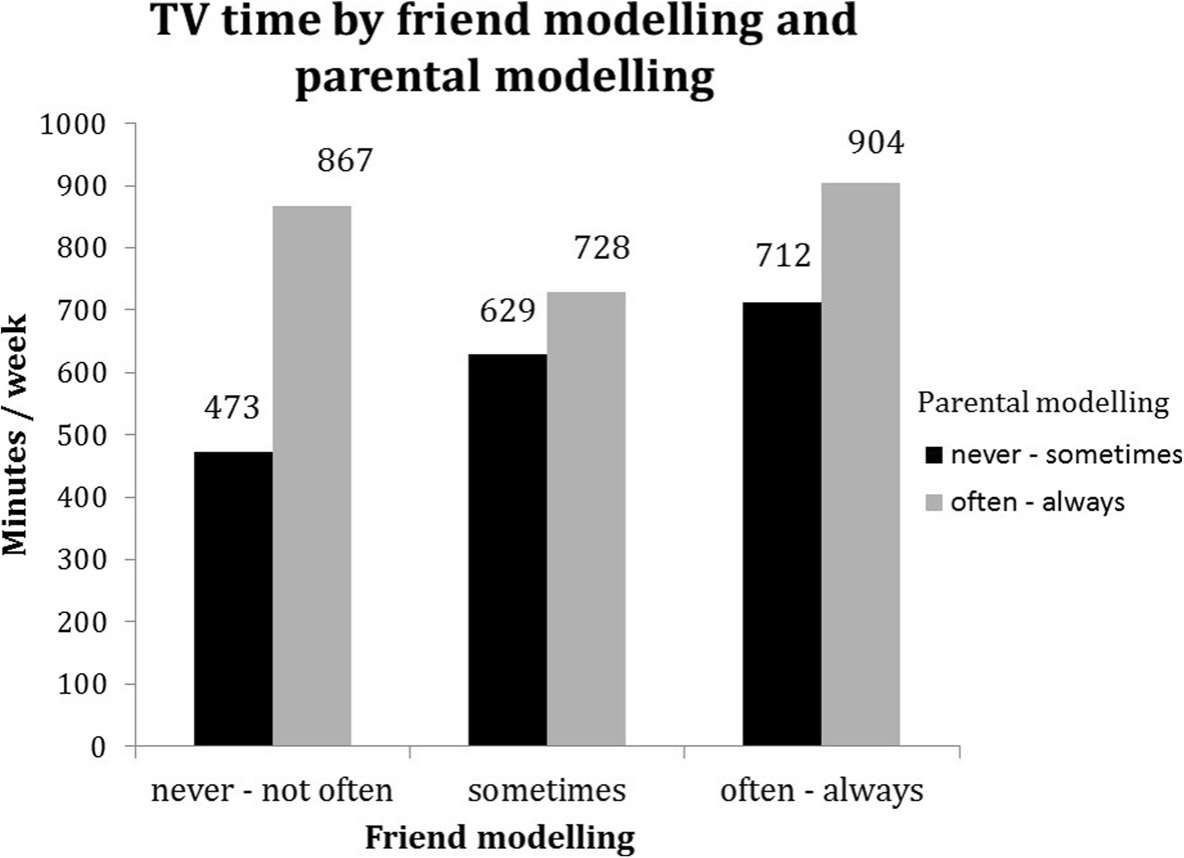
Moderation by parental modelling in the association between friend modelling and TV time.

**Figure 9:**
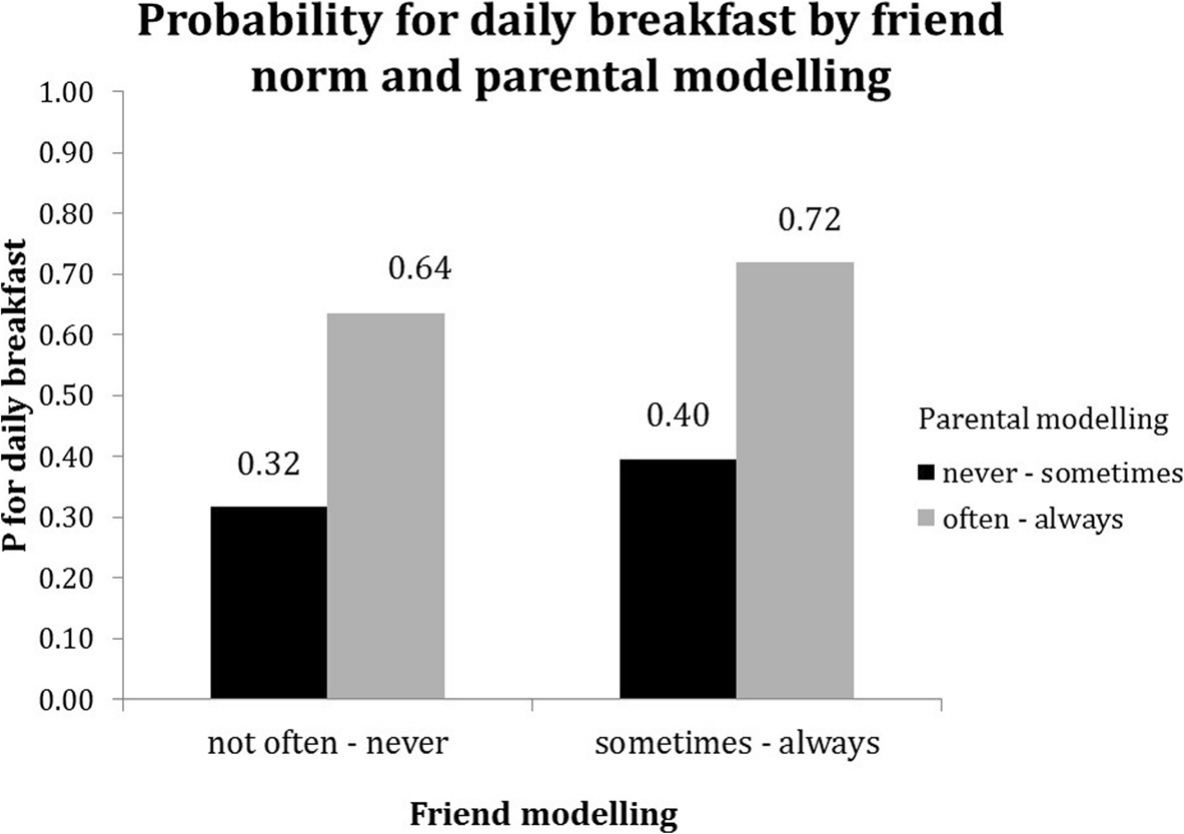
Moderation by parental modelling in the association between friend modelling and daily breakfast.

Stratified analyses for TV time suggest that friend modelling was more strongly associated with TV time in case of low parental modelling (Table 5). Figure 8 illustrates that in the high parental modelling stratum (grey bars) TV time was in general higher than in the low parental modelling stratum (black bars), and that high friend modelling was also linearly related to more TV time in the stratum of low parental modelling, but not in the stratum of high parental modelling.For daily breakfast consumption stratified analyses suggest that low friend modelling is stronger associated with daily breakfast in case of high parental modelling. Figure 9 further illustrates that this might be due to a relatively high probability for daily breakfast in the high parental modelling stratum (grey bars). Figure 9 further illustrates that despite this, the highest probabilities for daily breakfast were observed in the stratum of high parental modelling.

## Discussion

The current study assessed associations of and interactions between friend and parent influences with schoolchildren’s EBRBs across Europe. Results showed that both friend and parental norm and modelling were significantly associated with children’s EBRBs. In addition, we found evidence that friend and parental factors interact. In general this study showed that friend influences were stronger when no family rules were in place. Furthermore, the combination of unfavourable friend and unfavourable parent influences was associated with high soft drink consumption, while the combination of favourable friend and parent influences was associated with a higher probability for daily breakfast consumption.

Previous studies and reviews already indicated that social influences are important for EBRBs in youth [9,44,45], and that these social influences can have different forms and sources [21,34,46]. However, only a few studies looked at parental and friend factors simultaneously [25,30-34]. In contrast to findings of Kirby et al. [25], in our sample parental influences remained significantly associated with EBRBs in a multivariate model that included the friend influences. Although the study by Kirby et al. involved the same age group as the current study, it is more often found that in primary schoolchildren parents do influence physical activity levels of their children. A review by Edwardson and Gorely [47] found that parents played an important role in children aged 9–11 years especially by being active role models. This association was less clear for adolescents aged 12–18 years [47], also in line with the conclusion of the review by Uijtdewilligen and colleagues who found insufficient evidence for the prospective association between parental PA and adolescent’s PA [48]. The study sample of the ENERGY-project is in the transition to adolescence, but it is still in the age group where parents are expected to have a regulatory role which may explain the significant association of family rules, parental norms and modelling with their children’s EBRBs, even when friend influences are taken into account.

In addition, the current study suggests that parental and friend influences interact and may strengthen or weaken each other’s effect. Salvy et al. suggested that parents may have an inhibitory influence on unhealthy eating habits [31]. In our study we also found that favourable parental factors (i.e. rules in place, parents thinking that drinking soft drink is bad and low parental modelling) tempered the association of unfavourable friend norms and high friend modelling with high soft drink intake. It may be that having family rules in place reflects a more restrictive and controlling parenting style such as an authoritative or authoritarian parenting style, and that in families with authoritative or authoritarian parenting styles others outside the family system have less influence.

Furthermore, also for daily breakfast consumption, we found that if children reported high parental modelling, they were very likely to consume breakfast daily, even if they perceived unfavourable friend norms and friend modelling. That parental norms and modelling were stronger associated with daily breakfast consumption than friend norms and modelling seems plausible, as children usually do not have breakfast together with their friends. In addition, we found that friend norms and modelling were associated with sport participation and TV time. However, significant interaction between parental and friend influences was found in 10 out of the 20 potential interactive associations, indicating that associations of friend norms and modelling with EBRBs might depend on the parental norms and modelling. The current study included a selection of potential behavioural correlates and the strength of associations and interactions may depend on what variables are studied. In the current study the clearest pattern was found for the interaction between family rules and friend modelling. This may be explained by the fact that having family rules in place was a clearer concept for the participating children than parental modelling or parental norms, resulting in more valid answers (as shown in [40]) and thus a more straightforward pattern.Nevertheless, in general our results indicate that in this age group parental as well as friend influences are important for engagement in specific EBRBs and that both joint effects and moderating effects are important (Figure 1) depending on the specific parent and friend factors and behaviour.

Social influences on human behaviour and health have been well established and are an important topic for further study in social psychology and health promotion research [34,49]. Leading theories of determinants of health behaviour such as Social Cognitive Theory [20], the Theory of Planned Behaviour [50] and social-ecological models of health behaviour [51,52] all presume that social influences such as norms and/or modelling are important determinants of health behaviours. However, interactions between such influences from different ‘sources’, i.e. in the present study parents and friends, has not been studied before. If the present results are supported in further research, such interactions should be taken into account in theory and models of determinants of health behaviour for youth.

The major strength of the current study is its sample size. Furthermore, this is one of the first studies looking at interactions between parent and friend influences on four different EBRBs. A limitation of the study is its cross sectional design which restrains drawing conclusion on the direction and causality of the relationships. As previously suggested [53] it may very well be that parents react on their children’s behaviours by setting rules and initiating favourable example behaviour and communicating favourable norms and beliefs. Furthermore, children may seek friends that are similar to them, also with respect to EBRBs and beliefs regarding the EBRBs [54]. Finally, the current study used self-reported data, which may suffer from recall bias and social desirability. However, previously assessed reliability of the items was mostly good – excellent. In addition, validity of the EBRB items was mostly good to excellent, except for the items assessing norms and modelling. The latter is most likely due to the general difficulty to assess construct validity of cognitions, especially in this age group, and the lack of a gold standard.

Taking these limitations into account, the results suggest that both parental as well as friends norms and modelling are associated with engagement in EBRBs among 10–12 year-olds, and that parental norms, modelling and rules may also moderate friends’ influences. Therefore, parental as well as friend norm setting and modelling should be considered when planning health promotion interventions. These results confirm the importance of involving parents in school-based interventions [55]. More insight is still needed in how parental and friend involvement can be improved, how modelling and norm setting occurs and how this can be used to promote a healthy energy balance.

## Conclusion

Parental and friends norm and modelling are associated with schoolchildren’s energy balance-related behaviours. Having family rules or showing favourable parental modelling and norms seems to reduce the potential unfavourable associations of friends’ norms and modelling with the EBRBs.

## Abbreviations

EBRBs: Energy balance-related behaviours; ICC: Intra class correlation.

## Competing interests

The authors declare that they have no competing interests.

## Authors’ contributions

JB, SJtV, MJMC, IDB, LM and YM designed the international study. JB supervised the international study. All authors contributed to the development of measurement protocols and instruments and coordinated and supervised the data collections in the participating countries. SJtV conceived the manuscript and conducted the analyses. All authors assisted in the interpretation of the results. SJtV drafted the manuscript. JB and MJMC provided input on an early draft and all authors critically reviewed subsequent drafts. All authors approved the submitted manuscript.
